# Supplementary material for: Analysis of PPARγ Signaling Activity in Psoriasis
Source: Int J Mol Sci. 2021 Aug 10;22(16):8603. doi: 10.3390/ijms22168603 (PMC8395241; doi:10.3390/ijms22168603)
Supplement: Supplementary file 1 [file ijms-22-08603-s001.zip › Supplemental materials_Analysis of PPARg signaling activity in psoriasis/Pathway models/Models images and html files/PPARG inhibits phenotypes assotiated with psoriasis/104884.html]

multiple sclerosis


# Disease multiple sclerosis

|  |  |
| --- | --- |
| URN | urn:agi-meshdis:Multiple%20Sclerosis |
| Total Entities | 10 |
| Connectivity | 6285 |
| Name | multiple sclerosis |

---

|  |  |
| --- | --- |
| ChildConcepts | progressive relapsing multiple sclerosis |
|  | primary progressive multiple sclerosis |
|  | secondary progressive multiple sclerosis |
|  | acute relapsing multiple sclerosis |
|  | Schilder's disease |
|  | chronic progressive multiple sclerosis |
|  | relapsing-remitting multiple sclerosis |
|  | tumefactive multiple sclerosis |
|  | Balo's concentric sclerosis |
|  | Marburg acute multiple sclerosis |

---

|  |  |
| --- | --- |
| Pathway | Proteins with Altered Expression in Aging |
|  | Genes with Mutations Associated with Aging in Humans |
|  | Proteins Involved in Multiple Sclerosis |
|  | PPARG inhibits phenotypes assotiated with psoriasis |
|  | Neighbors of multiple sclerosis |

---

|  |  |
| --- | --- |
| MedScan ID | 9016132 |

---

|  |  |
| --- | --- |
| Alias | Sclerosis, Disseminated |
|  | Sclerosis, Multiple |
|  | sclerosis multiplex |
|  | Generalized Multiple Sclerosis |
|  | Ms (Multiple Sclerosis) |
|  | Multiple Sclerosis |
|  | Disseminated Sclerosis |
|  | Multiple scleroses |
|  | Insular Sclerosis |
|  | Multiple Scelrosis |

---

|  |  |
| --- | --- |
| MeSH ID | D009103 |
|  | M0014197 |

---

|  |  |
| --- | --- |
| MeSH Heading | Multiple Sclerosis |

---
